# Efficacy and feasibility of a humor training for people suffering from depression, anxiety, and adjustment disorder: a randomized controlled trial

**DOI:** 10.1186/s12888-019-2075-x

**Published:** 2019-03-20

**Authors:** Nektaria Tagalidou, Eva Distlberger, Viola Loderer, Anton-Rupert Laireiter

**Affiliations:** 1 Department of Psychology, University of Salzburg, Hellbrunner Straße 34, 5020 Salzburg, Austria; 2 Faculty of Psychology, University of Vienna, Liebiggasse 5, 1010 Vienna, Austria

**Keywords:** Humor training, Depression, Anxiety disorder, Adjustment disorder, RCT

## Abstract

**Background:**

Humor trainings have positive effects on mental health and well-being. However, studies investigating the effects of humor trainings in clinical samples are still rare. This study investigated the efficacy and feasibility of a humor training for people suffering from depression, anxiety and adjustment disorders.

**Methods:**

Based on a diagnostic interview (SCID I and II), 37 people were randomized into a training (*n* = 19) or wait list control group (*n* = 18) and completed questionnaires at pre, post, and 1 month follow-up. After the training group had completed its training and evaluation measures, the wait list control group received the training and the outcomes of the group were additionally evaluated (post2 and follow-up2).

**Results:**

After training, improvements in humor-related outcomes were observed for the training group, but these were relativized when compared to the wait list control group. Secondary outcomes remained unaffected by the training. In addition, the training group reported interpersonal difficulties. Within-group analyses of the wait list control group after completion of their training showed effects on almost all primary and secondary outcomes and feedback indicated a better atmosphere.

**Conclusions:**

In summary, the different outcomes of the two groups are surprising and can show potential moderators of efficacy, such as interpersonal and group-specific climate variables. Since moderators of humor trainings in clinical samples have not been investigated at all, future studies should consider integrating them into their design.

**Trial registration:**

The study was retrospectively registered in the German Clinical Trials Register (DRKS00012443) on May 16, 2017.

## Background

Mental disorders show high prevalence rates [1] in today’s society and represent a significant burden for those affected and the health care system [2]. Research into their treatment is crucial and most approaches, such as pharmacological or psychotherapeutic treatment, have already proven their effectiveness [3, 4]. In general, recovery from mental illness focuses mainly on reducing psychopathology. However, current trends in rehabilitation research attempt to integrate new facets into recovery, such as the promotion of well-being, life satisfaction or positive affectivity in general [5]. Research shows that the integration of new forms of treatment, especially those that promote positive aspects such as personal resources, well-being, or positive emotions, can bring a sustained improvement in mental health. However, there is still much unknown and further research is urgently needed [6]. As already postulated by the broaden-and-built theory, the experience of positive affect acts as an upward spiral and builds personal resources and resilience, while depression is alleviated [7–10]. Therefore, the combination of reducing psychopathology and increasing positivity can complement standard treatment and create a more holistic approach to mental health.

In addition to the already established interventions [7, 9], a new and promising way to promote positive emotions is humor. The use of humor is positively related to well-being [11–13], serves as an emotion regulation strategy [14–16], and can be an efficient tool for dealing with negative life situations [17–20]. Humor also has positive effects on other aspects of our daily lives, such as social or physical functioning [21]. Humor can have a significant impact on human flourishing. Its broad applicability in various areas of life makes it an effective tool for sustainable happiness and well-being. However, people with mental disorders seem to have problems integrating and applying humor into their daily lives and therefore miss the opportunity to benefit from it. Depression seems to have a negative influence on the cognitive and affective understanding of humor [22], the exhilaration created by funny stimuli, and the ability to use humor as a coping strategy [23]. Furthermore, people with alcoholism [24] and schizophrenia [25–27] also show different impairments in cognitive and affective aspects of humor. In addition, gelotophobia, defined as the fear of being laughed at by others [28, 29], appears to be more pronounced in people with social phobia or cluster A personality disorders [30] and is positively associated with introversion, neuroticism and poorer emotional regulation skills [30–33]. Deficits in humor are likely to be found in people with mental disorders, especially depression, and can limit its positive effects on mental health and well-being. To overcome this problem, recent research explores how to promote humor and thus developes various interventions, with humor training being the most popular one [13]. Humour trainings are usually conducted in groups, last several weeks and focus on different topics of humour behaviour. They have shown effectiveness in non-clinical samples by increasing positive affect and life satisfaction and reducing depression, anxiety or stress [34–36]. Humor trainings were also sporadically tested in clinical samples and have shown the same promising results: studies on depressed elderly patients have shown that satisfaction with life, resilience and cheerfulness has improved [37, 38]. Similar results were found in a study with inpatients suffering from depression [39]. There was an improvement in cheerfulness and coping humor (i.e. the ability to use humor to cope with stress) and a reduction in seriousness. However, the results should be interpreted with caution as the sample size was very small and there was no control group. The latest study investigating the effects of humor trainings was conducted with inpatients suffering from schizophrenia [40]. A randomized controlled trial showed that the training was effective in many outcome variables, such as the negative and positive symptoms of schizophrenia, depression, anxiety, and humor-related constructs such as coping humor or appreciating humor.

### Aims and research questions

As mentioned earlier, experiencing positive emotions has a positive impact on mental health and humor is an effective way to promote positive emotions. However, people with mental disorders seem to have deficits in applying humor and experiencing cheerfulness, thus could benefit positively from humor trainings. Research investigating this hypothesis is relatively scarce as few studies have implemented the training in clinical settings. In addition, the effects of humor trainings in clinical settings have been studied only for inpatients with depression or schizophrenia. The effects on people suffering from other mental disorders who are treated on an outpatient basis have not been studied at all. The study bridges this gap and examines the efficacy and feasibility of a humor training for people with various common mental disorders. In addition, the study increases ecological validity by offering the training in an outpatient setting, as more patients are treated outpatient than inpatient. We decided to investigate three common mental disorders that could benefit from the promotion of humor and positive emotions: Depression, anxiety disorders and adjustment disorder. Depression was integrated into the design of the study as it is mainly characterized by negative affectivity and anhedonia [41]. Promoting positive emotions could be a helpful tool against this negative affectivity. Adjustment disorder was included as one of the main symptoms is also depressive mood and thus has a pathology pattern similar to depression [42]. Anxiety disorders were integrated as humor can be an effective strategy for reducing non-clinical and clinical anxiety [43–47]. The outcomes of the study were divided into primary and secondary ones: First, we wanted to assess if the humor training increases humor-related outcomes that are directly related to the training (coping humor and cheerfulness). Second, we wanted to test if the training can also affect mental health related outcomes linked to participants’ symptoms (depression, anxiety, well-being, and gelotophobia). Although studies on the efficacy of humor trainings have been reported, studies on the feasibility and applicability of the trainings are rare. Therefore, the study also includes feasibility assessments to get a deeper overview of the training. Lastly, a major problem of the research on humor trainings in clinical settings is the methodological limitations of the studies [48]. This study attempts to overcome these methodological deficits by implementing a randomized controlled design with a wait list control group.

## Methods

### Design

The study’s design was a randomized controlled trial, in which groups were randomized by selecting a true random number application (www.random.org) followed by a balanced randomization (1:1) to either the training or wait list control group. The training group received the intervention first and both groups completed evaluation questionnaires simultaneously (pre-treatment, post-treatment, and at a 1 month follow-up after completing the training by the training group). After the training group had finished its training and both groups had completed evaluation questionnaires, the wait list control group began their training. Due to the obvious interpersonal and cohesion problems in the training group, and to get a better overview of the efficacy and feasibility of the training in general, we decided to evaluate the results of the wait list control group in a single-arm trial design additionally. We shared the same questionnaires after they had finished their training (post-treatment2) and again 1 month after its completion (follow-up2). The humor training was conducted in the outpatient clinic of the Department of Psychology of the University of Salzburg. The study was approved by the ethics commission of the University of Salzburg (44/2016) and registered in the German Clinical Trials Register (DRKS00012443).

### Participants

To be able to conduct statistical analyses between the groups and their interaction, the sample size was estimated in advance with G*Power 3.1 [49]. The calculations were carried out with a medium effect size of *f* = 0.25, a power of *β* = 0.80, and an α level of 0.05. A total sample size of 28 was proposed. When considering dropout, however, it is evident that a higher sample size is required. McDermut, Miller, and Brown [50] report an average attrition rate of 18.6% for group therapies of mood disorders. Based on these results, a total N of at least 33 participants had to be recruited and the final sample size for the study was 37. Recruitment was made via a promotional article in the Salzburg local newspaper and the recruitment phase took place between January 2017 and February 2017. The most important inclusion criterion for participating in the study was the presence of at least one of the following mental disorders: (recurrent) depressive disorder, currently mild or moderate, anxiety disorder, or adjustment disorder. In addition, good language skills and no cognitive deficits were required. Mental disorders excluded from the study were: severe (recurrent) depression, posttraumatic stress disorder, personality disorders, bipolar disorder, schizophrenia, current substance abuse, and delusional, manic, or psychotic episodes in the past (alone or co-occurring with the target diagnoses). People were allowed to continue their psychological and pharmacological treatments while participating in the training.

### Procedure

Recruitment for this study took place parallel to a second humor training study in the same institution [51]. Neither studies nor recruited samples overlap (they are independent) as the participants in this sample had an acute mental disorder, while the second study included participants who had only subclinical symptoms (i.e. no diagnosed mental disorder). Interested persons registered on the training homepage via a contact form. Then, they were contacted by telephone for a quick pre-screening concerning their general motivation for participation and organisational issues. Persons who seemed suitable for participation were invited for a face-to-face diagnostic interview. Three independent outpatient clinic employees conducted the interviews with the Structured Clinical Interview for DSM-IV (SCID I and II) [52]. They were all undergoing training as clinical psychologists and already had sufficient experience in clinical diagnostics. If people met the inclusion criteria and had no observable cognitive or linguistic deficits in the diagnostic interview, they were invited for training and were randomized to one of the groups. Before the training began, informed consent and a non-disclosure agreement were signed. Although the humor training focuses mainly on humorous and positive aspects of the participants’ daily lives, a non-disclosure agreement had to be signed and adhered to in order to protect the participants’ privacy (e.g. when someone talked about personal experiences or difficulties) and thus build a basis of trust within the groups. There were two training groups and two wait list control groups of eight to ten people each. The group sessions took place on the same weekdays and at the same time both in the training and wait list condition (Tuesday and Thursday evening). The group leaders were the same for all four groups. Two Master’s students, who were at the end of their studies, conducted the humor trainings together (two trainers per group). They had sufficient experience with clinical psychology and group therapy and were intensively trained in the manual by the study leader. In addition, they were supervised by the study leader and an experienced clinical psychologist and had to record each session to check manual adherence.

### Humor training

For our study we used the manual by Falkenberg, McGhee and Wild [53], which is a German humor training specifically developed for people with mental disorders. The sessions build on each other, starting with simpler topics such as “finding humor in everyday life” and ending with more advanced topics such as “laughing at personal weaknesses”. All sessions were aiming at the same goal: learning different strategies to be able to cope everyday stressors with humor and increasing positive affect. Together with the trainers, we adapted the programme and made minor modifications to make it more suitable for our participants (e.g. including more modern media), but the basic structure of the training remained unchanged (see Table 1).

**Table 1:** Topics of the humor training

Session	Content
1. Session	Introduction
2. Session	Playfulness (vs. seriousness)
3. Session	Laughter and its positive influences
4. Session	Verbal humor
5. Session	Humor in everyday life
6. Session	Personal weaknesses
7. Session	Humor and stress

In detail, our manual consisted of seven sessions held once a week for 90 min. Each session dealt with a specific topic. In general, the sessions contained psychoeducational elements and various exercises as role-plays and games to train humor. Between sessions, participants had to do homework to practice what they had learned. In addition, a humor diary was introduced, which the participants could fill in voluntarily. Here, they were asked to write down three funny things they had experienced during the day every evening. Studies show that a humor diary is effective in increasing happiness and reducing depression [54–56], so it was included in the training.

### Outcome measures

Outcome measures were based on self-report questionnaires and completed online. Both groups received the questionnaires at pre-treatment, post-treatment, and 1 month follow-up the end of training of the training group. Additionally, the wait list control group received the same questionnaires again at the end of their own training (post-treatment2) and 1-month later (follow-up2). At the end of the training, a voluntary feedback questionnaire was distributed to both groups.

#### Primary outcomes

The *Coping Humor Scale (CHS)* assesses the participant’s ability to use humor to cope with difficult life events [18]. The scale was translated into German and modified as a state-sensitive questionnaire asking about coping humor in the past 2 weeks and includes seven items with a four-point Likert-scale ranging from one to four. Internal consistency (Cronbach’s α) was α = .72 (pre-treatment). As another primary outcome, the German State-Trait-Cheerfulness-Inventory *(STCI)*, in the state version, measures the current amount of exhilaration [57, 58]. The questionnaire contains 30 items with a four-point Likert-scale (one to four) and is divided into three subscales: cheerfulness, seriousness, and bad mood. Internal consistencies for the three subscales were α = .94, α = .83, and α = .94 (pre-treatment).

#### Secondary outcomes

The German *Center for Epidemiological Studies Depression Scale Revised (CESD-R)* estimates the amount of depression in the past 2 weeks [59]. The scale includes 15 items on a four point Likert-scale (zero to three) resulting in a total sum score of maximum 45. A cut-off score of > 17 indicates the existence of depressive symptoms in non-clinical as well as clinical samples [59]. Internal consistency was α = .90 (pre-treatment). The German *State-Trait-Anxiety-Inventory (STAI)* in the state version measures the current amount of anxiety [60]. The questionnaire contains 20 items with a four-point Likert scale (one to four) resulting in a total sum score of maximum 80. Cut-off scores are not available for the state version as it was designed to be a change sensitive outcome, which cannot be compared to representative samples [60]. Internal consistency was α = .93 (pre-treatment). The German *WHO-5 Well-Being Index (WHO-5)* is a screening tool for subjective well-being in the past 2 weeks [61]. It includes five items on a six-point Likert-scale (zero to five) resulting in a total sum score of maximum 25. A cut-off score of < 13 indicates low well-being and the possibility of having depressive symptoms [61]. Its internal consistency was α = .83 (pre-treatment). Gelotophobia was measured by the German *Gelotophobia-Questionnaire (GELOPH-15*), which assesses the habitual fear of being laughed at by others [28, 29]. The questionnaire contains 15 items with a four-point Likert-scale (one to four). A mean of ≥2.5 indicates a slight degree of gelotophobia. Internal consistency was α = .88 (pre-treatment).

Finally, a self-generated feedback questionnaire evaluated feasibility and satisfaction with the training. Fourteen quantitative items and three open questions capture positive and negative aspects of the training, and suggestions for improvement.

### Statistical analyses

The analyses were calculated using IBM SPSS Statistics 24 [62]. Differences in demographics were analyzed using Chi-square test for categorical data and independent samples t-test with Bonferroni correction for continuous data. Efficacy of the training was analyzed using intention-to-treat technique (ITT) without imputation method. Instead, linear mixed models (LMM) with restricted maximum likelihood estimation (REML) and compound symmetry as covariance type were calculated. LMMs were preferred in the evaluation of longitudinal data because they can overcome the problem of missing data without imputation [63]. Outcomes were checked for normal distribution using Shapiro-Wilk test and were normally distributed, expect for coping humor and seriousness (only for pre-treatment). However, all other outcomes showed normal distribution at all times. The main effects of group and time, and group by time interactions were considered in the interpretation of results. In addition, post hoc tests with Bonferroni correction were calculated to analyse the differences between the training and control group. Effect sizes of post hoc tests were analysed considering estimated marginal means and reported in Pearson *r* [64]. Cohen [65] categorizes *r* = 0.10 as small effect, *r* = 0.30 as medium effect, and *r* = 0.50 as large effect. The evaluation of the wait list control group was based on the first follow-up as pre-measure2, post-treatment2, and follow-up2. Again, LMMs were calculated and no imputation technique for missing data was used. Additionally, post hoc tests with Bonferroni correction were calculated and Pearson *r* interpreted (based on estimated marginal means). Finally, the feedback questionnaire was analyzed descriptively for quantitative items and a qualitative analysis with MAXQDA for open questions was performed. The responses of participants were rated and categorized by two independent psychologists in an inductive way. Interrater agreement for the categories was fair to moderate ranging between κ = 0.39–0.74.

## Results

### Sample characteristics

**Fig. 1:**
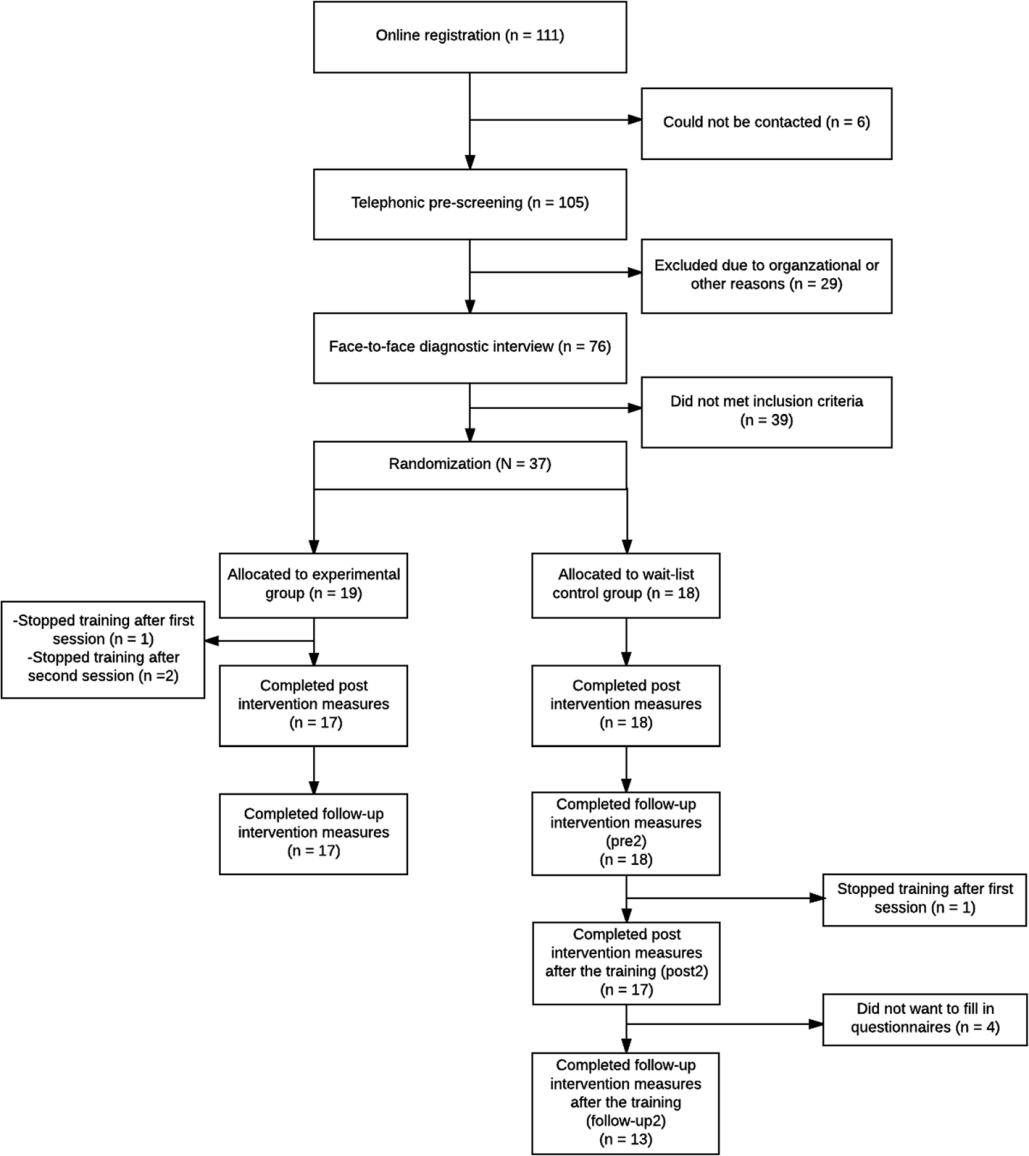
Flowchart of the study

A total of 111 people initially registered on the training website (for this and the subclinical study [51]), 105 of whom were contacted by telephone for pre-screening. Seventy-six (68.5%) individuals were eligible to participate in the face-to-face diagnostic interview. In the end, 37 (33.3%) individuals met the inclusion criteria for participating in this study and were randomized to either the training (*n* = 19) or wait list control group (*n* = 18). A detailed description of the selection procedure can be found in the flowchart (see Fig. 1).

The participants were predominantly female (*n* = 27, 73.0%) and had Austrian citizenship (*n* = 32, 86.5%). They were between 24 and 76 years old with an average age of 50.86 (*SD* = 13.68). Their educational level was high, as 26 participants (70.3%) had a 12-year education and 23 persons (62.2%) were employed or were studying. A total of 22 people had (recurrent) depression (59.5%), eight had an adjustment disorder (21.6%) and seven had an anxiety disorder (18.9%). Seven participants (18.9%) were in current psychotherapy and six participants (16.2%) regularly took psychotropic drugs. There were no differences in demographic variables between the training and wait list control group (see Table 2). In addition, there were no differences in baseline measures between the two groups. During the study period, four persons changed their (external) treatment: one person changed their medication at the end of training, two persons started psychotherapy (one at the end of training and one at of follow-up), and one person completed psychotherapy at the end of training.

**Table 2:** Demographic characteristics of the sample (*N* = 37)

	TG (*n* = 19) M (SD) or n (%)	CG (*n* = 18) M (SD) or n (%)	Statistics
Age, M (SD)	48.68 (14.38)	53.17 (12.90)	t_(35)_ = − 1.00, *p* = .326
Gender, n (%)			
Female	13 (68.4%)	14 (77.8%)	χ^2^_(1, N = 37)_ = 0.41, *p* = .522
Male	6 (31.6%)	4 (22.2%)	
Nationality, n (%)			
Austrian	18 (94.7%)	14 (77.8%)	χ^2^_(1, N = 37)_ = 2.28, *p* = .132
Other (German, Swiss)	1 (5.3%)	4 (22.2%)	
Education, n (%)			
≥ 9 years of education (compulsory school)	5 (26.3%)	5 (27.8%)	χ^2^_(3, N = 37)_ = 1.22, *p* = .748
≥ 12 years of education (A level)	3 (15.8%)	2 (11.1%)	
≥ any tertiary education (e.g. university)	11 (57.9%)	10 (55.6%)	
Not specified	0 (0.0%)	1 (5.6%)	
Employment, n (%)			
Currently employed	7 (36.8%)	11 (61.1%)	χ^2^_(5, N = 37)_ = 4.18, *p* = .524
Retirement	5 (26.3%)	4 (22.2%)	
Sick leave	2 (10.5%)	0 (0.0%)	
Parental leave/ Educational leave	1 (5.3%)	1 (5.6%)	
Student	3 (15.8%)	2 (11.1%)	
Not specified	1 (5.3%)	0 (0.0%)	
Primary diagnoses, n (%)			
(Recurrent) depression Anxiety disorder	13 (68.4%) 1 (5.3%)	9 (50.0%) 6 (33.3%)	χ^2^_(2, N = 37)_ = 4.76, *p* = .092
Adjustment disorder	5 (26.3%)	3 (16.7%)	
Treatment, n (%)			
Psychotherapy Psychotropic drugs	2 (10.5%) 4 (21.1%)	5 (27.8%) 2 (11.1%)	χ^2^_(1, N = 37)_ = 1.41, *p* = .235

Notes: *TG* training group, *CG* Wait list control group; * *p* < .05, ** *p* < .01, *** *p* ≤ .001

### Primary outcomes

The ITT analysis showed no significant group by time interaction in any outcome: Coping humor (*F*_(2,63.92)_ = 2.08, *p* = .133), cheerfulness (*F*_(2,64.15)_ = 0.60, *p* = .942), seriousness (*F*_(2,65.02)_ = 1.57, *p* = .217), and bad mood (*F*_(2,62.45)_ = 0.73, *p* = .488). Furthermore, significant main effects of time were found for all primary outcomes indicating changes in both groups irrespective of treatment: Coping humor (*F*_(2,63.92)_ = 10.33, *p* ≤ .001), cheerfulness (*F*_(2,64.15)_ = 3.94, *p* = .024), seriousness (*F*_(2,65.02)_ = 16.37, *p* ≤ .001), and bad mood (*F*_(2,62.45)_ = 11.34, *p* ≤ .001). Going more into detail, there were significant within-group changes for the training group from pre to post and pre to follow-up in coping humor, seriousness, and bad mood with medium to large effect sizes; however, the wait list control group showed changes in these outcomes too. Between-group analyses, therefore, did not indicate significant changes between the two groups at post and follow-up (s. Table 3 for descriptive statistics and effect sizes).

**Table 3:** Observed means (OM), observed standard deviations (OSD), estimated means (EM), standard error (ER), and effect sizes (Pearson r) for the outcome measures of training and wait list control group (*N* = 37)

	n	Pre		Post		Follow-up		Pre-post within	Pre-FU within	Post between	FU between
		OM (OSD)	EM (SE)	OM (OSD)	EM (SE)	OM (OSD)	EM (SE)				
CHS											
Training group	19	2.19 (0.57)	2.17 (0.10)	2.54 (0.60)	2.51 (0.10)	2.46 (0.59)	2.43 (0.10)	0.57***	0.41*	0.15	0.01
Wait list control group	18	2.23 (0.54)	2.23 (0.12)	2.41 (0.53)	2.41 (0.12)	2.43 (0.46)	2.43 (0.12)	0.33	0.36		
Cheerfulness (STCI)											
Training group	19	2.02 (0.52)	2.00 (0.11)	2.36 (0.63)	2.35 (0.11)	2.27 (0.72)	2.26 (0.11)	0.31	0.17	0.06	0.02
Wait list control group	18	2.08 (0.41)	2.07 (0.13)	2.41 (0.61)	2.41 (0.12)	2.28 (0.45)	2.28 (0.12)	0.32	0.21		
Seriousness (STCI)											
Training group	19	3.38 (0.40)	3.41 (0.14)	2.83 (0.62)	2.84 (0.14)	2.74 (0.70)	2.75 (0.14)	0.53**	0.59***	0.04	0.16
Wait list control group	18	3.31 (0.29)	3.29 (0.11)	2.90 (0.59)	2.90 (0.11)	2.98 (0.43)	2.99 (0.11)	0.52**	0.44*		
Bad mood (STCI)											
Training group	19	2.66 (0.58)	2.74 (0.19)	2.06 (0.86)	2.09 (0.20)	2.11 (0.93)	2.13 (0.20)	0.58***	0.56**	0.03	0.08
Wait list control group	18	2.64 (0.70)	2.62 (0.18)	2.01 (0.60)	2.01 (0.17)	2.30 (0.81)	2.30 (0.17)	0.45*	0.26		
Depression (CES-D)											
Training group	19	18.63 (9.21)	18.63 (2.30)	13.82 (9.75)	14.15 (2.41)	15.24 (10.81)	15.56 (2.41)	0.30	0.21	0.13	0.01
Wait list control group	18	17.39 (8.53)	17.39 (1.67)	17.28 (6.40)	17.28 (1.67)	15.67 (6.13)	15.67 (1.67)	0.01	0.19		
Anxiety (STAI)											
Training group	19	50.11 (12.51)	50.11 (3.38)	45.06 (15.00)	45.44 (3.54)	45.89 (16.55)	46.27 (3.54)	0.20	0.17	0.11	0.13
Wait list control group	18	50.61 (11.51)	50.61 (2.60)	49.94 (11.15)	49.94 (2.60)	51.56 (10.43)	51.56 (2.60)	0.04	0.05		
Well-Being (WHO-5)											
Training group	19	13.42 (4.03)	13.42 (1.02)	15.35 (4.27)	15.39 (1.08)	15.29 (5.03)	15.33 (1.08)	0.25	0.24	0.08	0.05
Wait list control group	18	13.33 (3.85)	13.33 (0.91)	16.39 (3.58)	16.39 (0.91)	14.78 (4.11)	14.78 (0.91)	0.57***	0.31		
Gelotophobia (GELOPH)											
Training group	19	2.25 (0.61)	2.25 (0.15)	1.94 (0.63)	1.97 (0.15)	2.03 (0.69)	2.07 (0.15)	0.32	0.22	0.03	0.03
Wait list control group	18	2.18 (0.64)	2.18 (0.15)	1.94 (0.62)	1.94 (0.15)	2.13 (0.69)	2.13 (0.15)	0.41*	0.10		

*Note: CHS* Coping Humor Scale, *STCI* State-Trait-Cheerfulness-Inventory – state version, *CES-D* Center for Epidemiological Studies-Depression Scale, *STA*: State-Trait-Anxiety-Inventory – state version, WHO-5 WHO-5 Well-Being Index, *GELOPH* Gelotophobia questionnaire, † *p* ≤ .10, * *p* < .05, ** *p* < .01, *** *p* ≤ .001

### Secondary outcomes

Again, the ITT analysis revealed no significant group by time interaction for any outcome: Depression (*F*_(2,64.86)_ = 1.18, *p* = .315), anxiety (*F*_(2,66.53)_ = 0.56, *p* = .575), well-being (*F*_(2,66.61)_ = 0.53, *p* = .593), and gelotophobia (*F*_(2,66.75)_ = 0.28, *p* = .754). Main effects of time were found for well-being (*F*_(2,66.61)_ = 5.69, *p* = .005) and gelotophobia (*F*_(2,66.75)_ = 4.74, *p* = .012). Depression and anxiety showed no effects at all. Post hoc tests did not show any significant effects for the training group from pre to post or pre to follow-up. Furthermore, effect sizes ranged only from small to medium and effect sizes of the wait list control group were also small. However, for well-being a significant and large effect size from pre to post emerged (*r* = 0.57), which diminished in the follow-up (*r* = 0.31). Also, for gelotophobia a medium effect from pre to post was found (*r* = 0.41), which also disappeared in the follow-up (*r* = 0.10). A complete overview of the results can be found in Table 3.

### Feedback of the training group

Table 4 summarizes the results of the feedback questionnaire. Although the effects of the training were small, overall satisfaction with the training (rated on a scale from one to five) was good (*M* = 3.92, *SD* = 0.76). In summary, three people (23.1%) would definitely recommend the training, nine people (69.2%) would probably recommend it and only one person would not recommend it (7.7%). Items concerning the interest and comprehensibility of the content were rated as good to very good. The applicability of the contents in everyday life was moderate to good (*M* = 3.62, *SD* = 1.04) and the improvement of symptoms (*M* = 3.00, *SD* = 1.08) and cheerfulness (*M* = 3.08, *SD* = 1.19) moderate. Open format questions were analysed using a semantic content analysis for each question separately. There was a substantial interrater agreement (*κ* = 0.62) for the positive aspects of the training. People mostly liked group related variables as interaction/ exchange/ atmosphere within the group (*n* = 8), and the trainers (*n* = 2). Regarding the negative aspects of the training, there was a substantial interrater agreement (*κ* = 0.74), too. People mostly criticized group variables as interaction/ exchange/ atmosphere (*n* = 4). In addition, people mentioned lack of time (*n* = 2), trainers (*n* = 2), and dropout of participants (*n* = 2) as a critical point. Lastly, missing aspects/ recommendations for the future showed a fair interrater agreement (*κ* = 0.39). Participants mostly wanted more time (*n* = 3). A detailed list of all answers can be found in Table 5.

**Table 4:** Results of the feedback questionnaire for the training (*n* = 13) and wait list control group (*n* = 12)

	Training group M (SD)	Wait list control group M (SD)
Overall, I was satisfied with training.	3.92 (0.76)	4.67 (0.89)
The contents of training have been understandable.	4.46 (0.78)	4.75 (0.62)
The addressed topics have been interesting.	4.23 (0.73)	4.67 (0.89)
The structure of sessions had a logical and plausible order.	4.23 (0.73)	4.75 (0.45)
The discussions about humor topics have been interesting.	3.92 (1.04)	4.67 (0.89)
The humor topics have been useful for me.	4.08 (0.86)	4.58 (0.67)
I liked the games within training.	4.08 (0.95)	4.42 (1.00)
The mixture of theory and practice was good.	4.00 (1.16)	4.58 (0.67)
The location was comfortable.	3.69 (0.95)	3.75 (1.36)
I think I can transfer the learned into my everyday life.	3.62 (1.04)	4.33 (0.99)
After training, I can integrate humor better in my everyday life.	3.85 (1.14)	4.42 (0.79)
After training, I experience more cheerfulness than before.	3.08 (1.19)	4.33 (0.65)
I think my problems became lower because of training.	3.00 (1.08)	4.08 (0.79)
Would you recommend the training?^a^	3.40 (0.65)	3.67 (0.65)

*Note*: 1 = does not apply at all, 2 = hardly applies, 3 = applies partly, 4 = fairly applies, 5 = applies completely; ^a^ 1 = No, in no case, 2 = Rather not, 3 = Rather yes, 4 = Yes, in any case. Items of the feedback questionnaire were also used in Tagalidou et al. [51]

**Table 5:** Number of codings for the open format questions regarding feedback of participants in the training (*n* = 13) and wait list control group (*n* = 12)

	Training group	Wait list control group
*Positive aspects*		
Group (interaction/ exchange/ atmosphere)	8	6
Trainer	2	7
Content of the training	2	5
Transfer to daily life	1	2
Games and exercises	3	2
Effects of the training	2	2
*Negative aspects*		
Group (interaction/ exchange/ atmosphere)	4	1
Time	0	3
Trainer	2	0
Dropout of participants	2	0
Exercises	2	0
Too much “happyology”	1	0
Premises	0	1
Group size	0	1
*Missing/ Recommendations for the future*		
More time	3	6
Laughter yoga	0	2
More thematic depth	0	2
Better compliance of participants	1	0
More jokes	1	0
More practice	1	0
Integrating humor into nature	1	0
Better trainer leadership	0	1
Better premises	0	1
Booster sessions	0	1

### Outcomes of the wait list control group

Due to the participants’ criticism of the inharmonic atmosphere and the unusually small effects of the intervention in the training group, we wanted to deepen the efficacy evaluations of the humor training and thus also analysed the results and the feedback questionnaire of the wait list control group. LMMs showed main effects of time for nearly all outcomes: coping humor (*F*_(2,27.46)_ = 15.45, *p* ≤ .001), cheerfulness (*F*_(2,29.91)_ = 10.66, *p* ≤ .001), bad mood (*F*_(2,28.74)_ = 5.50, *p* = .010), depression (*F*_(2,29.28)_ = 7.08, *p* = .003), anxiety (*F*_(2,28.98)_ = 16.30, *p* ≤ .001), well-being (*F*_(2,29.39)_ = 3.62, *p* = .039), and gelotophobia (*F*_(2,28.36)_ = 9.75, *p* ≤ .001). Only seriousness showed a trend for the main effect of time (*F*_(2,30.84)_ = 2.84, *p* = .074). Regarding effect sizes of post hoc tests, primary outcomes as coping humor, cheerfulness, and bad mood showed significant medium to large effect sizes at post2 and follow-up2 (*r* between 0.45 and 0.70). Seriousness showed a trend from pre to post (*r* = 0.47), which disappeared in the follow-up (*r* = 0.27). Secondary outcomes also changed in the desired direction with anxiety showing the largest effects from pre2 to post2 (*r* = 0.68) and pre2 to follow-up2 (*r* = 0.66). Depression, well-being, and gelotophobia showed medium to large effect sizes at post2 and follow-up2, too (*r* between 0.45 and 0.58). Table 6 gives a complete overview of the descriptive statistics and effect sizes for the wait list control group. Lastly, the feedback from the participants was very positive. The satisfaction with the training was very good (*M* = 4.67, *SD* = 0.89) and nine people (75.0%) would definitely recommend it. Only two people (16.7%) would probably recommend it and only one person would not recommend it (8.3%). In general, the training was rated very positively in terms of interest and comprehensibility, as well as the subjectively perceived improvement in symptoms. For a detailed description of the feedback see Table 4. Again, open format questions were qualitatively analysed and the interrater agreement on positive, negative, and missing aspects was already mentioned above. The responses show that the participants liked the trainers (*n* = 7), group variables as interaction/ exchange/ atmosphere within the group (*n* = 5), and the content of the training (*n* = 5). The most important negative aspect was the lack of time (*n* = 3). Missing aspects/ recommendations for the future were more time (*n* = 6), including laughter yoga (*n* = 2), and more thematic depth (*n* = 2). See Table 5 for further results.

**Table 6:** Observed means (OM), observed standard deviations (OSD), estimated means (EM), standard error (ER), and effect sizes (Pearson r) for the outcome measures of the wait list control group (*n* = 18)

	Pre		Post		Follow-up		Pre-post within	Pre-FU within
CHS	2.43 (0.46)	2.43 (0.12)	2.97 (0.47)	2.93 (0.12)	2.95 (0.53)	2.89 (0.13)	0.70***	0.63***
Cheerfulness (STCI)	2.28 (0.45)	2.28 (0.13)	2.86 (0.57)	2.85 (0.13)	2.81 (0.61)	2.76 (0.14)	0.63***	0.52**
Seriousness (STCI)	2.98 (0.43)	2.98 (0.12)	2.64 (0.55)	2.64 (0.12)	2.75 (0.54)	2.73 (0.14)	0.39†	0.27
Bad mood (STCI)	2.30 (0.81)	2.30 (0.17)	1.81 (0.62)	1.85 (0.17)	1.81 (0.67)	1.82 (0.19)	0.47*	0.45*
Depression (CES-D)	15.67 (6.13)	15.67 (1.52)	11.65 (6.24)	11.58 (1.54)	11.23 (7.61)	11.43 (1.65)	0.52**	0.50*
Anxiety (STAI)	51.56 (10.43)	51.56 (2.32)	41.00 (9.43)	41.28 (2.37)	41.31 (9.69)	40.92 (2.56)	0.68***	0.66***
Well-Being (WHO-5)	14.78 (4.11)	14.78 (1.01)	17.12 (4.36)	17.13 (1.03)	16.62 (4.75)	16.19 (1.11)	0.45*	0.26
Gelotophobia (GELOPH)	2.13 (0.69)	2.13 (0.15)	1.75 (0.57)	1.78 (0.15)	1.87 (0.65)	1.76 (0.16)	0.58**	0.57**

*Note: CHS*: Coping Humor Scale, *STCI*: State-Trait-Cheerfulness-Inventory – state version, *CES-D*: Center for Epidemiological Studies-Depression Scale, *STAI*: State-Trait-Anxiety-Inventory – state version, WHO-5: *WHO*-5 Well-Being Index, *GELOPH*: Gelotophobia questionnaire, † *p* ≤ .10, * *p* < .05, ** *p* < .01, *** *p* ≤ .001

## Discussion

Three things are surprising about the results of the training: First, although within the training large effects on humor-related outcomes were observed, secondary outcomes were unaffected by the training. This does not correspond to the results of previous research, where effects were also found in non-humor-related constructs [37–40]. Second, the improvements in humor-related outcomes within the training group were relativized by simultaneous changes in the wait list control group, although they received no treatment while waiting. Third, after training, the control group showed greater effects (especially in secondary outcomes) than the training group. We will discuss these unexpected results and try to find possible explanations for these inconsistencies. The secondary outcomes (depression, anxiety, well-being, and gelotophobia) did not change significantly within the training group. These results contradict existing research that has shown that humor affects mental health related outcomes. For example, depression reduced by participating in various humorous interventions [34, 39, 54–56, 66, 67]. In addition, humor has proven to be an effective strategy against non-clinical and clinical anxiety [43–47] and is associated with well-being [12]. One possible explanation for the small effects could be that participants did not devote as much attention to the humor training as recommended. Although changes in the humor outcomes could be observed, the commitment to training could have been too low to cause changes beyond the humor outcomes. The training group had problems with the inharmonic group constellation and the absence of some participants (negative aspect group/dropout: *n* = 6), while the control group did not have these difficulties (negative aspect group/dropout: *n* = 1). These assumptions are also consistent with the trainers’ perceptions. In order to better evaluate the training, the trainers had to monitor each session and record all problems and conflicts. They also recognised the interpersonal problems of the training groups and discussed them with their supervisor. Although some problems could be solved, the general inharmonious atmosphere within the sessions could not be completely eliminated. Humor is a true social phenomenon that depends on people’s positive and friendly interactions and mutual acceptance [21]. However, a generally negative atmosphere is an antagonist of this phenomenon and could have contributed to an impaired learning that could have subsequently reduced the outcomes of the training. Group cohesion and alliance have an important influence on the treatment outcomes of group therapies [68–70], and future research should consider evaluating moderating variables such as these in humor trainings. There is no study that has investigated the influence of interpersonal problems on humor trainings, but it would be interesting to do so. The second question is the fluctuation in the outcomes of the wait list control group. Although some effects of the intervention were found in the training group, they were relativized because the control group showed similar changes while waiting. In particular, humor-related constructs showed significant changes with partially large effects. One explanation for these unusual results might be that participants waiting for treatment can generally improve in their outcomes, whether through spontaneous remission or re-test effects [71–74]. However, the large changes in this sample tend to exceed the effects reported in these studies. Another additional explanation for the results may be that participants in the wait list control group had positive expectations and were motivated and interested in humor as they waited for their training to begin. Therefore, they could have dealt with humorous topics or at least be more receptive regarding humor in everyday life. Positive expectation and hope effects are unspecific factors in psychotherapy and psychological interventions in general and can contribute significantly to the improvement of symptoms [75]. A combination of both spontaneous remission/re-test effects and positive expectation effects can explain the significant fluctuations in results during waiting. However, no assessments were made to support this hypothesis. Future research should consider the evaluation of expectation and hope effects in the wait list control group to control possible fluctuations and inexplicable changes. The third question about the results is the strong change in the outcomes of the control group after they had received their own training. Almost all primary and secondary outcomes were positively influenced and showed significant effects. Coping humor and cheerfulness changed from pre2 to post2 and pre2 to follow-up2 with medium to large effect sizes. In contrast to the training group, the secondary outcomes also changed, with anxiety showing the greatest effects and a longer decline until follow-up2. In addition, the feedback questionnaire was evaluated very positively and the participants were more satisfied with the training compared to the training group. It is striking that the answers to the open format questions were more benevolent and also more positive, as fewer negative aspects and more positive aspects were reported. The participants praised the other group members and the only point of criticism was the lack of time for more intensive group discussions. These results were in line with the trainers’ expectations, who reported a more cheerful and positive atmosphere in the wait list control group during the training. There were fewer conflicts and participants were usually more motivated and engaged in the training. The positive results and feedback of the wait list control group compared to the mediocre results of the original training group reinforce the assumption that a positive atmosphere between participants plays an important role in the efficacy of the training. However, one question remains unanswered: Why were the two groups so different in terms of interpersonal and motivational aspects?

A possible explanation can be found in the constellation of the groups. As can be seen in Table 2, the wait list control group had more people with anxiety disorders than the training group, which had more people with depression and adjustment disorder. As mentioned earlier, depression is characterized by anhedonia and lack of cheerfulness. In addition, depressive people show an impairment of social functioning [76, 77] and general humor deficits [22]. The reported difficulties could have contributed to a more difficult atmosphere and lack of cohesion in the training group, which later also affected the commitment to intervention.

Future studies should investigate humor trainings in homogeneous groups of mental disorders and not in mixed groups. In this way, it would be possible to test whether and how the training differs in terms of efficacy and feasibility for different mental disorders.

In summary, the inconsistent results of the training and control group were surprising and raised questions about future research on humor trainings: Do interpersonal variables moderate the efficacy of the training? Do expectation and hope effects influence the participants of the wait list control group? Is it possible that some mental disorders (e.g. depression) counteract with humorous interventions? Future research is definitely needed to get a comprehensive picture of the efficacy of humor trainings and moderating/confounding variables should also be considered.

### Limitations

First, for organisational reasons it was not possible to carry out a follow-up of more than 1 month, both in the regular study and in the additional evaluation of the wait list control group. It would have been interesting to test the stability of the outcomes over a longer period, as the outcomes of the training in the wait list control group remained quite stable after completion of the training. Second, the sample size was rather small, although many people had initially registered for the training. However, since the inclusion criteria were very strict, many people had to be rejected after the face-to-face diagnostic interview. In order to study larger samples, a longer recruitment phase with more time for advertising and diagnostic assessment would be necessary. Third, blinding of participants was not possible and participants in the wait list control group were informed that the training group had started the intervention while they were waiting. Due to the delayed start, the control group could have been more motivated and have more intensive expectations of the training and its results than the training group.

Fourth, the results were based only on self-reporting. Due to the lack of suitable instruments, no external valuations could be made, although it would have been interesting to consider also ratings from related parties. Fifth, the sample was based on self-selection, as the subjects decided to participate on their own if they had interest. This could have led to a selection bias (e.g. more extraverted persons registering for the group training). Sixth, it should be noted that differences in outcomes between this and previous studies can be explained by different methods and patient samples. This study is an RCT with a mixed patient sample (depression, anxiety and adjustment disorder). Previous studies were quasi-experimental or had uncontrolled designs that primarily treated homogeneous groups of participants. Therefore, the comparability of the results is limited.

## Conclusions

The humor training showed inconsistent results. The training group changed mainly in humor-related outcomes, but these changes did not go beyond the outcomes of the wait list control group. In addition, the feedback was mediocre and indicated problems in the atmosphere and cohesion of the group. There were strong differences in the outcomes of the wait list control group after their training. Efficacy of the training was high in almost all outcome variables and the feedback was positive, especially with regard to the group constellation. This study raised questions regarding moderating process and relationship variables in humor trainings, which should be systematically investigated in future research.

## Abbreviations

CESD-R: Center for Epidemiological Studies Depression Scale Revised
CG: Control group
CHS: Coping humor scale
EM: Estimated means
ER: Standard error
GELOPH-15: Gelotophobia-Questionnaire
ITT: Intention-to-treat
LMM: Linear mixed models
OM: Observed means
OSD: Observed standard deviations
RCT: Randomized controlled trial
REML: Restricted maximum likelihood estimation
SCID: Structured Clinical Interview for DSM-IV
STAI: State-trait-anxiety-inventory
STCI: State-trait-cheerfulness-inventory
TG: Training group
WHO-5: WHO-5 Well-Being Index
